# Impact of inflammatory signaling on radiation biodosimetry: mouse model of inflammatory bowel disease

**DOI:** 10.1186/s12864-019-5689-y

**Published:** 2019-05-02

**Authors:** Sanjay Mukherjee, Evagelia C. Laiakis, Albert J. Fornace, Sally A. Amundson

**Affiliations:** 1 Center for Radiological Research, Columbia University Irving Medical Center, New York, NY 10032 USA; 2 Department of Oncology, Lombardi Comprehensive Cancer Center, Georgetown University, Washington, DC 20057 USA; 3 Department of Biochemistry and Molecular & Cell Biology, Georgetown University, Washington, DC 20057 USA

**Keywords:** Inflammatory bowel disease (IBD), Biodosimetry, Gene expression, Inflammation, Mouse model

## Abstract

**Background:**

Ionizing Radiation (IR) is a known pro-inflammatory agent and in the process of development of biomarkers for radiation biodosimetry, a chronic inflammatory disease condition could act as a confounding factor. Hence, it is important to develop radiation signatures that can distinguish between IR-induced inflammatory responses and pre-existing disease. In this study, we compared the gene expression response of a genetically modified mouse model of inflammatory bowel disease (*Il10*^*−/−*^*)* with that of a normal wild-type mouse to potentially develop transcriptomics-based biodosimetry markers that can predict radiation exposure in individuals regardless of pre-existing inflammatory condition.

**Results:**

Wild-type (WT) and *Il10*^*−/−*^ mice were exposed to whole body irradiation of 7 Gy X-rays. Gene expression responses were studied using high throughput whole genome microarrays in peripheral blood 24 h post-irradiation. Analysis resulted in identification of 1962 and 1844 genes differentially expressed (*p* < 0.001, FDR < 10%) after radiation exposure in *Il10*^*−/−*^ and WT mice respectively. A set of 155 genes was also identified as differentially expressed between WT and *Il10*^*−/−*^ mice at the baseline pre-irradiation level. Gene ontology analysis revealed that the 155 baseline differentially expressed genes were mainly involved in inflammatory response, glutathione metabolism and collagen deposition. Analysis of radiation responsive genes revealed that innate immune response and p53 signaling processes were strongly associated with up-regulated genes, whereas B-cell development process was found to be significant amongst downregulated genes in the two genotypes. However, specific immune response pathways like MHC based antigen presentation, interferon signaling and hepatic fibrosis were associated with radiation responsive genes in *Il10*^*−/−*^ mice but not WT mice. Further analysis using the IPA prediction tool revealed significant differences in the predicted activation status of T-cell mediated signaling as well as regulators of inflammation between WT and *Il10*^*−/−*^ after irradiation.

**Conclusions:**

Using a mouse model we established that an inflammatory disease condition could affect the expression of many radiation responsive genes. Nevertheless, we identified a panel of genes that, regardless of disease condition, could predict radiation exposure. Our results highlight the need for consideration of pre-existing conditions in the population in the process of development of reliable biodosimetry markers.

**Electronic supplementary material:**

The online version of this article (10.1186/s12864-019-5689-y) contains supplementary material, which is available to authorized users.

## Background

Chronic inflammation is a part of many human diseases [1]. Studies reveal that approximately 5–7% of the western population suffers from some sort of chronic inflammatory condition [1, 2]. Ionizing radiation is a known pro-inflammatory agent [3]. Thus, pre-existing inflammatory disease could potentially confound radiation biodosimetry approaches used for medical triage during a radiological event.

The inflammatory aspect of radiation has been studied extensively in recent years [4–7]. Radiation induced inflammation has previously been reported in the atomic bomb survivors [8]. Lately, there has been renewed interest in the role of inflammation in radiotherapy [5, 9]. On one hand, radiation-mediated immunotoxicity is one of the limiting factors for radiotherapy while on the other hand, radiation facilitated increased immunogenicity is being used to enhance tumor treatment [9–11]. Various factors govern the degree of inflammation after radiation exposure. Genetic background of an individual and preexisting disease conditions can greatly affect the outcome of a radiation exposure [12–14].

Any accidental or intentional large-scale radiological event will result in a large number of individuals requiring medical attention, which will require an effective biodosimetry tool for efficient distribution of medical resources [15, 16]. An individual with an underlying inflammatory condition could show heightened immune responses to radiation exposure and thus obscure some biodosimetry methods. Thus, it is necessary to consider the immune modulating effects of radiation and the potential impact of underlying inflammatory conditions in the development of effective biodosimetry tools for triage purposes.

Various biodosimetric methods have been proposed and evaluated for large-scale radiation exposure scenarios [16]. Gene expression represents an emerging approach to biodosimetry, and could provide estimates of absorbed dose and severity of radiation–induced injury [17–19]. It has been shown that genes involved in inflammation and immune response are among the strongest early responders following ionizing radiation exposure [18, 19]. In addition, radiation exposure triggers a long-lived inflammatory response, with up regulation of inflammatory markers and immune dysfunction that can persist for decades [3, 8].

In this study, we used *Il10*^*−/−*^, a mouse model of human inflammatory bowel disease (IBD), and compared its transcriptomic responses to radiation with those of normal wild-type (WT) mice. Interleukin (IL) 10 is a potent anti-inflammatory cytokine and plays a significant role in regulating the expression of Th1 cytokines, MHC mediated antigen presentation and antibody production from B-cells [20]. After radiation exposure, this cytokine plays an important role in regulating inflammation as well as free radical mediated damage in the neighboring non-exposed cells and tissues [21, 22]. In the absence of IL-10 cytokine, the *Il10*^*−/−*^ mice become highly susceptible to enteric bacterial pathogens, and show a heightened inflammatory response to pathogen infection [23, 24]. Similar to IBD patients, the *Il10*^*−/−*^ mice have been shown to have higher levels of IL23-producing macrophages, which through their interaction with circulating T-cells cause production of inflammatory cytokines [25]. In terms of radiation responses, *Il10*^*−/−*^ mice have been shown to readily develop colitis following a smaller dose of radiation compared to normal WT- mice [Unpublished data]. Thus, we have used this genotype to represent those individuals in the population who have an underlying chronic inflammatory condition that could potentially skew the results from gene expression based biodosimetry during mass triage after a radiation emergency. This study was undertaken as an initial investigation into the potential impact of chronic inflammation on gene expression based radiation biodosimetry markers.

## Methods

### Animals and irradiation

Wild-type (WT) C57BL/6 and *Il10*^*−/−*^ mice (B6.129P2-*Il10*^*tm1Cgn*^/J) were obtained from Jackson Laboratory and bred at Georgetown University as het x het. The study was conducted according to the Georgetown University Institutional Animal Care and Use Committee (IACUC). All the mice (including the WT) were housed in the same room with similar bedding, food, and water under specific pathogen- free (SPF) housing conditions. In addition, the experiment was conducted in the same time for all mice, minimizing the potential of microbiome alterations in the facility. Furthermore, no other mice besides the ones in this study were housed in that room during the time of the experiment. Male littermates between 8 and 10 weeks of age were whole body irradiated with 7 Gy X-rays using an X-Rad 320 X-ray machine (Precision X-ray Inc., Brandford, CT). Sham-irradiated control mice (0 Gy) were treated the same as the exposed animals except the source was not switched on. The dose was selected based on the LD_50/30_ value of WT-C57BL/6 mice (background strain for *Il10*^*−/−*^mice), of 8Gy for X-ray irradiation [26]. It was reduced slightly (7 Gy) as the *Il10*^−/−^ mice appeared in early experiments to be more sensitive to radiation. Mice were euthanized 24 h after irradiation using carbon dioxide inhalation followed by cervical dislocation, per IACUC standard procedures. Approximately ~ 0.4 ml blood was collected from each mouse using cardiac puncture method, into a 15 ml centrifuge tube containing 1.6 ml PAXgene Blood RNA stabilization and lysis solution (PreAnalytix GmBH, catalog # 762165), and mixed thoroughly. The tubes were then frozen at − 80 °C, and shipped to Columbia University for RNA isolation. At the time of sacrifice, a drop of blood was also collected in EDTA Microtainer® tubes (BD medical, catalog #365974) for complete blood counts (CBC) using a Genesis hematology system (Oxford Science). Blood samples from 8 animals were used for CBC analysis.

### RNA isolation

Blood samples in PAXgene solution were allowed to reach room temperature for 2 h before proceeding to RNA isolation. RNA was purified following the PAXgene RNA kit recommendations with on-column DNase I treatment. Globin RNA was reduced using the Ambion GLOBINclear-mouse/rat kit (Thermo Fisher Scientific). RNA yields were quantified using the NanoDrop ND1000 spectrophotometer (Thermo Fisher Scientific) and RNA quality was checked by the 2100 Bioanalyzer (Agilent). High quality RNA with an RNA integrity number of at least 7.0 was used for microarray hybridization.

### Microarray hybridization

For both genotypes, RNA isolated from control and irradiated mouse blood samples (*n* = 5) was labeled with Cyanine-3 (Cy3) using the One-Color Low-Input Quick Amp Labeling Kit (Agilent Technologies, Santa Clara, CA) following manufacturer’s instructions. Study size (*n* = 5) was selected to provide 90% power to detect two fold changes, based on an analysis of our previous mouse microarray data using the methods of Lee and Whitmore [27]. A total of 100 ng of input RNA was used for each labeling process. The labeled RNA was purified using the RNAeasy mini kit (Qiagen). Purified RNA having a Cy3 specific activity more than 8 was used for microarray hybridization. A total of 1.65 μg of labeled RNA for each sample was fragmented and hybridized to mouse GE4x44 v2 microarrays (part no. G4846A; Agilent Technologies). The hybridization was carried out at 65 °C for 17 h in a hybridization oven followed by washing as recommended by the supplier (Agilent). Slides were scanned with the Agilent DNA microarray scanner (G2505B) and the images were analyzed with Feature Extraction software [v10.7] (Agilent) using default parameters for background correction and flagging non-uniform features.

### Microarray data analysis

Background corrected hybridization intensities were imported into BRB-ArrayTools, v. 4.3.2 (NCI, Biometric Research Branch, Bethesda, MD) [28] log2-transformed and median normalized. Non-uniform outliers or features not significantly above background intensity in 25% or more of the samples, as well as features not changing at least 1.5-fold in 20% or more of the samples were filtered out, giving 16,000 features that were used in subsequent analyses. The microarray data is available through the NCBI Gene Expression Omnibus (series no. GSE114142; http://www.ncbi.nlm.nih.gov/geo/query/acc.cgi?acc=GSE114142).

BRB-Array Tools was used to identify genes that were differentially expressed after radiation exposure in WT and *Il10*^*−/−*^ mice. Genes with *P* < 0.001 and having a false-discovery rate of less than 10% as calculated using the Benjamini-Hochberg method were considered significantly differentially expressed.

### Class prediction

We used class prediction methods in BRB-Array Tools to select genes and build predictors of radiation exposure status. The greedy pairs method [29] for feature selection was used to identify the 12 top-performing pairs of genes for discrimination between irradiated and control samples using the wild-type data as the training set. Seven classification methods (compound covariate predictor, linear discriminant analysis, 1- and 3-nearest neighbors, nearest centroid, support vector machines, and Bayesian compound covariate predictor) were used with the selected feature sets to predict the irradiation status of the remaining test samples (*Il10*^*−/−*^). The percentage correct classification was calculated for each approach. The same approach was then repeated using the *Il10*^*−/−*^ data as the training set and the wild-type data as the test set.

### Gene ontology and network analysis

The lists of differentially expressed genes were analyzed using the functional annotation tool of the Database for Annotation Visualization and Integrated Discovery (DAVID; v 6.7) [30]. Gene ontology terms and biological functions with a Benjamini-corrected *P* value < 0.05 were considered significantly over-represented within a gene list. Genes that were significantly differentially expressed in the two mouse strains were also imported into Ingenuity Pathway Analysis (IPA from QIAGEN Inc., www.qiagen.com/ingenuity) software and analyzed using IPA Core analysis and Comparative Analysis Tools. IPA uses curated information on the published relationships between gene products to predict networks and association between genes in a list. The upstream regulator analysis specifically uses information about the relationship between the activity of potential upstream regulatory factors and the expression changes of the measured genes to make predictions on the regulatory status of the upstream molecule. IPA generates a z-score for each factor in the upstream regulator analysis and for prediction of activation or inhibition state of biological functions. The IPA default cutoff of z ≥ 2 was used to predict activation and z ≤ − 2 to predict inhibition.

### Quantitative real-time RT-PCR

Real-time quantitative RT-PCR (qRT-PCR) was performed for selected genes using Taqman chemistry and the ABI 7900 Real Time PCR System. The Globin cleared purified RNA from 5 control and irradiated animals was used for cDNA synthesis using the High-Capacity cDNA Archive Kit (Life Technologies). Gene expression assays (primer/probe sets) were purchased from Thermo Fisher for the following genes: *Ifit3* (Mm00366278_m1), *Ifit1* (Mm00365614_m1), *Cdkn1a (*Mm04205640_g1), *Aen* (Mm00471554_m1), *Gstt1* (Mm00492506_m1), *Il18r1* (Mm00515178_m1), *Ccng1* (Mm00438084_m1), *Alas1* (Mm001235914_m1), *Acsbg1* (Mm00547366_m1), *Cyp2b6* (Mn00456591_m1), *Actb* (Mm00607939_s1). The ΔΔCT method was used to calculate expression relative to controls, using normalization to Actb expression. Each reaction was run in triplicate with 5 control and 5 irradiated samples, and means were compared using an unpaired t-test.

## Results

### Effect of radiation on blood cell counts of WT and *Il10*^*−/−*^ mice

In the present study, we compared gene expression in the peripheral blood of WT and *Il10*^*−/−*^ mice with and without exposure to ionizing radiation. As different blood cell types have different susceptibility to radiation, we first analyzed the effect of radiation on blood cell counts in the two genotypes 24 h post irradiation.

**Fig. 1:**
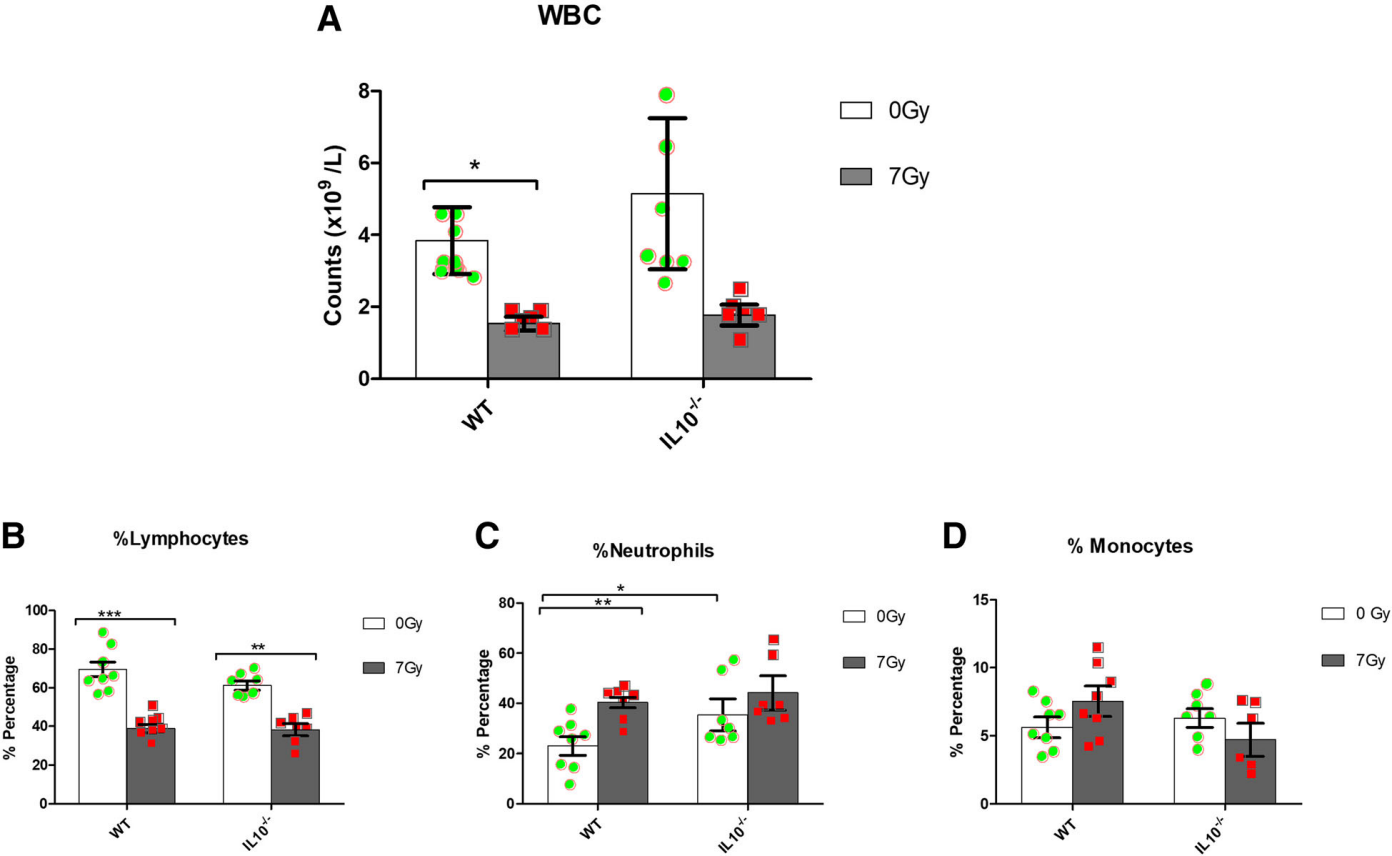
Total white blood cell count and percentages of different blood cell types in WT and *Il10*^−/−^ mice after 24 h of radiation exposure (7Gy). **a** Total WBC count **b** Lymphocyte (%) **c** Neutrophils (%) **d** Monocytes (%). Significant differences *P < 0.05; **P < 0.01; ***P < 0.001 (*n* = 8). Green circles (control) and red squares (irradiated) indicate values for individual animals of the respective genotype

The total number of white blood cells (WBC) and relative percentages of major blood cell population (i.e. lymphocytes, neutrophils and monocytes) is shown in Fig. 1. Overall, we found a decrease in WBC counts after radiation exposure, which was significant in WT, but not in *Il10*^*−/−*^*,* mice (Fig. 1a). Although post-irradiation WBC numbers were similar in both genotypes, the *Il10*^*−/−*^ controls showed a higher degree of variability. Analysis of different blood cellular components revealed significant decreases in the lymphocyte percentage in the blood of both WT and *Il10*^*−/−*^ mice after radiation exposure (Fig. 1b). There was an increase in neutrophil percentage 24 h after radiation exposure, which was statistically significant in WT mice (*P* < 0.01) but not in *Il10*^*−/−*^ mice (Fig. 1c). There was no significant difference in the blood monocyte percentages after radiation exposure in either genotype (Fig. 1d). Interestingly, we observed significant differences in the baseline percentage of neutrophils between WT and *Il10*^*−/−*^ mice pre-irradiation (Fig. 1c). In a separate experiment, we found that at a lower dose (2Gy) of radiation exposure, most of the decrease in the WBC counts had been recovered by Day 30 post irradiation in both genotypes (Additional file 1). We did not find any significant differences in red blood cell (RBC) and platelet counts after radiation exposure in WT or *Il10*^*−/−*^ mice.

### Baseline differences in gene expression between WT and *Il10*^*−/−*^ mice

**Fig. 2:**
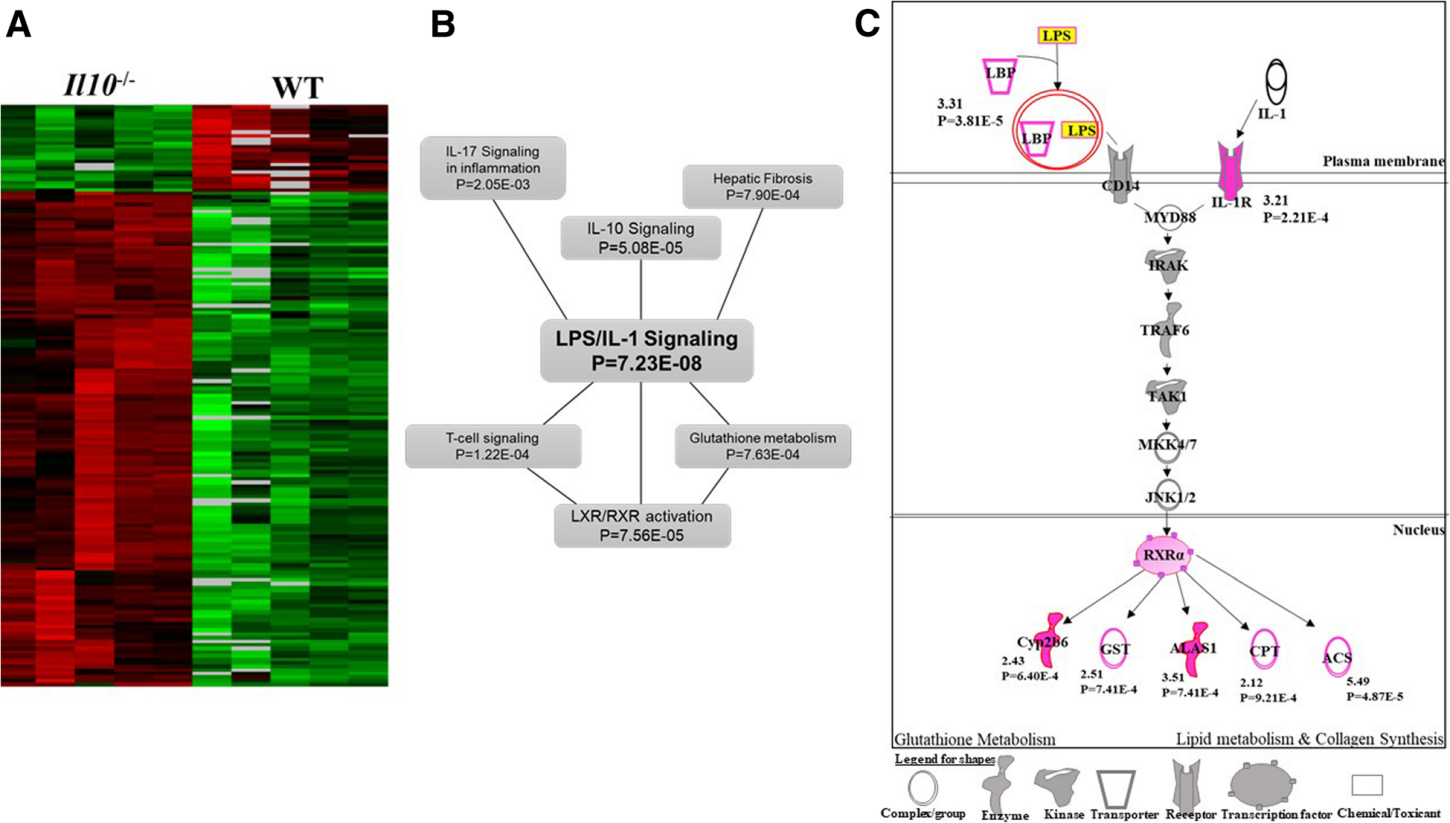
Analysis of 155 baseline differentially expressed genes between WT and *Il10*^−/−^ mice. **a** Heat map of expression of 155 genes. Annotation of genes is provided in Additional file 2A. **b** IPA based network analysis of significant (*P* < 0.05) canonical pathways (Additional file 2C) associated with the 155 genes **c** LPS/IL-1 Pathway. Genes identified as differentially expressed in *Il10*^−/−^ mice are highlighted in pink. The numbers below indicate fold change and *p*-value. LBP = LPS binding protein; Cyp2b6 = cytochrome P450 2B6; GST = Glutathione S-transferase; ALAS1 = Aminolevulinate synthase 1; CPT = Carnitine Palmitoyltransferase; ACS = Acetyl Coenzyme A synthetase

In order to detect baseline differences in gene expression between the two genotypes we performed class comparison analysis using BRB-ArrayTools [28]. The analysis identified 155 genes in *Il10*^*−/−*^ mice that were differentially expressed (*P* < 0.001; FDR < 10%) compared to the WT strain without radiation exposure (Table 1; Additional file 2A). Of these 155 differentially expressed genes, 120 (77%) had a significantly higher expression level in *Il10*^*−/−*^ mice, while 35 genes (23%) had a lower expression level compared to WT (Table 1) (Fig. 2a). Gene ontology analysis using DAVID [30] (Additional file 2B) showed that these genes were mainly involved in inflammatory response (Benjamini corrected *P* = 7.19 × 10^− 7^), glutathione metabolism (Benjamini corrected *P* = 1.38 × 10^− 5^) and collagen deposition (Benjamini corrected *P* = 2.16 × 10^− 4^).

**Table 1:** Differentially expressed genes in blood from WT and *Il10*^−/−^ mice 24 h post-irradiation (7Gy)

Class comparison	Number of differentially expressed genes^#^	Up-regulated genes	Down-regulated genes
*Il10*^−/−^ vs. WT^*^	155	120	35
Wild-type (WT) irradiated vs. control	1844	1093	751
*Il10*^−/−^ irradiated vs control	1962	994	968

^#^Statistical cut off: *P*-value< 0.001; FDR < 10%

^*^Genes differentially expressed in *Il10*^−/−^ mice compared to WT at baseline (0 Gy) without irradiation

Analysis using the IPA network analysis tool [QIAGEN Inc., www.qiagen.com/ingenuity] revealed that LPS (lipopolysaccharide)/IL-1 signaling was the most significantly enriched (*P* = 7.23 × 10^− 8^) canonical pathway in this 155 gene set (Fig. 2b; Additional file 2C). This represents a broad inflammatory response, as well as a central pathway linking other responses, including inflammation and glutathione metabolism, that were most affected in the gene ontology analysis of these genes (Fig. 2c). Some of these baseline genes were validated using real-time PCR and are represented in Additional file 3.

### Comparative analysis of gene expression response to radiation exposure between WT and *Il10*^*−/−*^ mice

**Fig. 3:**
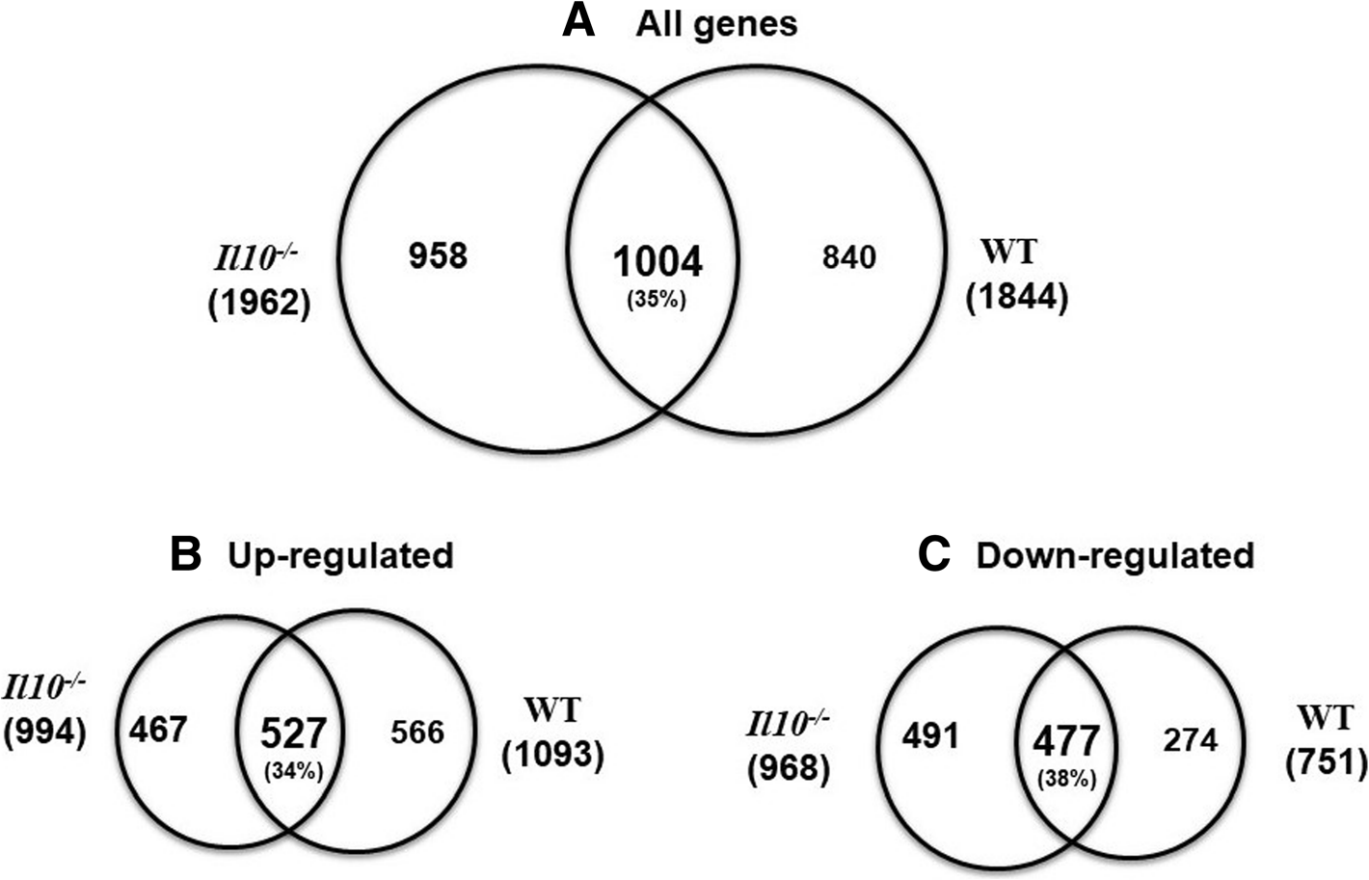
Differentially expressed genes **a**) Venn diagram showing overlap of the total number of differentially expressed genes (*P* < 0.001, FDR < 10%; Table 1) in WT and *Il10*^−/−^ mouse strains after 7Gy radiation exposure. There were 35% genes common between WT and *Il10*^−/−^ mice which showed significant expression change after radiation exposure. **b**) Represents overlap of the number of up-regulated genes and **c**) Represents overlap of the number of down-regulated genes after radiation exposure in the two genotypes

We next performed a comparative analysis of radiation responsive genes in WT and *Il10*^*−/−*^ mice. We identified a total of 2075 and 2158 differentially expressed features (*P* < 0.001, FDR < 10%) representing 1844 and 1962 known genes in WT and *Il10*^*−/−*^ mice respectively, after radiation exposure (Table 1; Additional file 4). In WT mice, 60% (1093 genes) of the differentially expressed genes were upregulated, and 40% (751 genes) were downregulated after radiation exposure. In *Il10*^*−/−*^ mice the differentially expressed genes were evenly distributed with 51% (994) up-regulated genes and 49% (968) downregulated genes (Table 1). Venn diagram analysis revealed 1004 (35%) radiation responsive genes common between WT and *Il10*^*−/−*^ mice out of which 527 were upregulated and 477 were downregulated (Fig. 3).

**Fig. 4:**
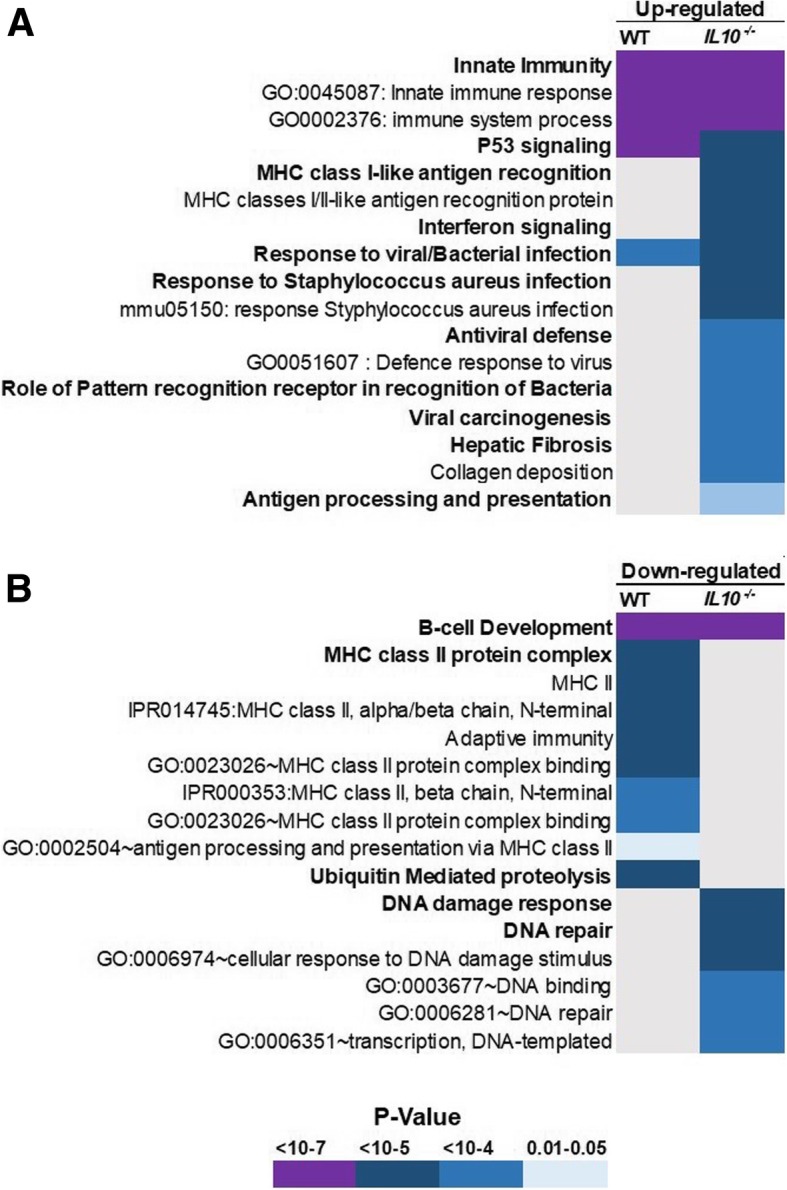
Gene Ontology (GO) Analysis. The DAVID resource tool was used to find overrepresented GO terms. GO terms with Benjamini-corrected *P* < 0.05 were considered statistically significant. The color of a cell represents a *P* value as indicated in the key. **a** Significant GO categories overrepresented in up-regulated genes in WT and *Il10*^−/−^ mice **b** Significant GO categories overrepresented among down-regulated genes in WT and *Il10*^−/−^ mice in response to radiation

Gene Ontology (GO) analysis with DAVID also revealed a level of similarity in terms of significant enrichment of radiation responsive gene ontology terms in WT and *Il10*^*−/−*^ mice (Fig. 4). We found innate immune response and p53 signaling were significantly enriched among radiation responsive genes in both WT and *Il10*^*−/−*^ mice (Fig. 4a). However, additional immune-related GO terms, such as MHC-I antigen presentation, interferon signaling, and hepatic fibrosis, were also enriched among up-regulated genes of *Il10*^*−/−*^ mice but not in WT after radiation exposure. Also, down-regulated genes in WT, but not *Il10*^*−/−*^, mice were significantly enriched with MHC-II protein complex terms, while DNA damage response and DNA repair terms were significantly enriched among down-regulated genes of *Il10*^*−/−*^ mice after radiation exposure (Fig. 4b).

**Fig. 5:**
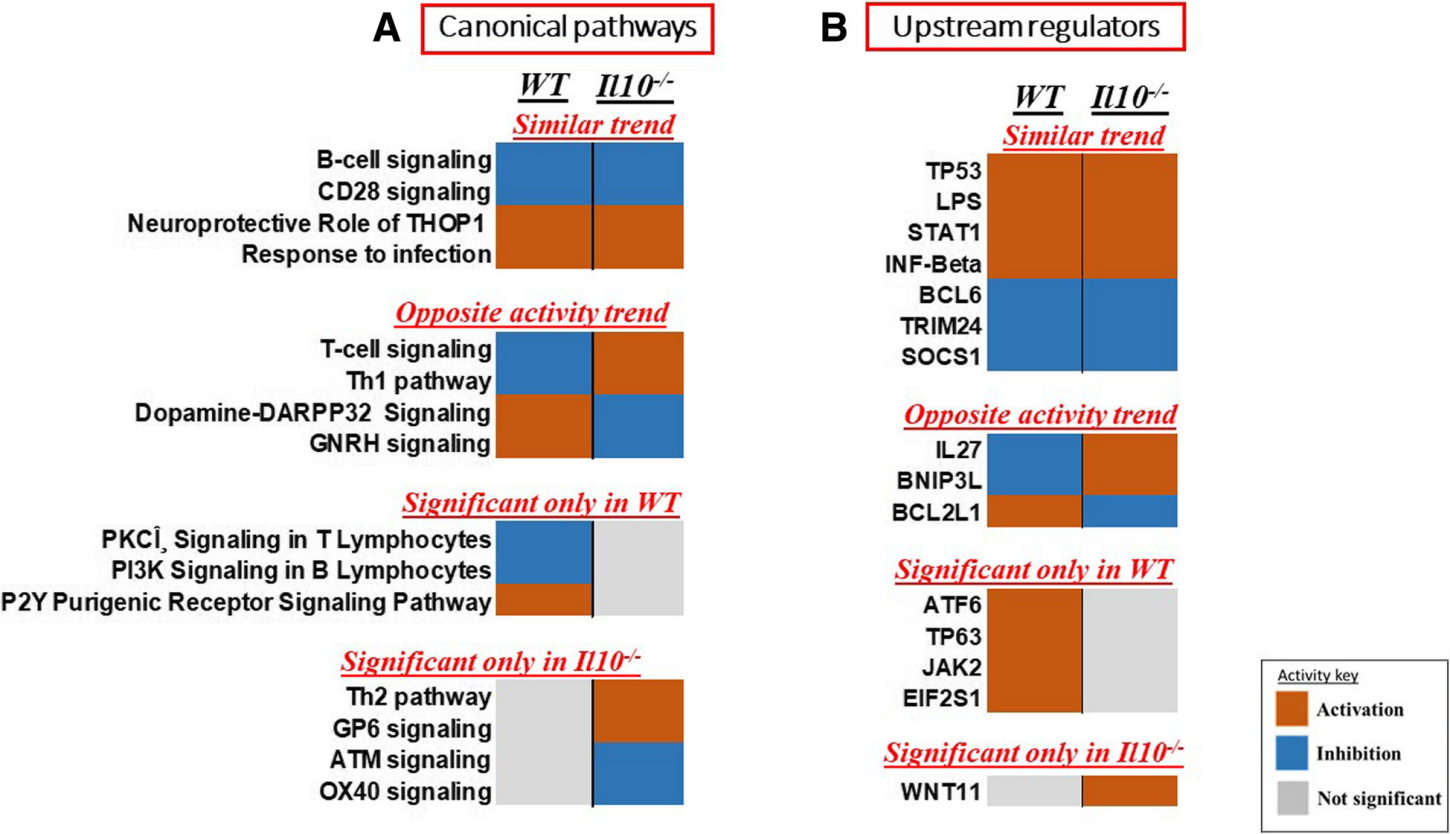
Comparative analysis of **a**) Canonical pathways and **b**) Upstream regulators responding to radiation in WT and *Il10*^−/−^ mice. IPA based statistical z-score was used to predict activation (z ≥ 2, orange) or suppression (z ≤ − 2, blue) of a particular pathway or regulator. Grey color indicates no significant activity

We then used the IPA comparison analysis tool to assess and compare categories of biological processes activated or inhibited in the two genotypes after radiation exposure, as predicted from gene expression changes. We used default z-score ≥ 2 for activation or z-score ≤ − 2 for inhibition as the significance cutoff for our analyses (Fig. 5). We found canonical pathways involved in immune response to infection and B-cell signaling showing similar trends of activity in the two genotypes after radiation exposure. However, the predicted activation status of T-cell signaling differed between the two genotypes after radiation exposure. The T-cell signaling pathway was predicted to be activated in *Il10*^*−/−*^ mice but inhibited in WT mice in response to radiation (Additional file 5). In addition, the Th-1 response, which is associated with inflammation, appeared to be activated in *Il10*^*−/−*^ mice but inhibited in WT mice. Hormone based signaling pathways also seemed to have different activity status after radiation exposure in the two genotypes (Fig. 5a).

We also performed a similar predictive analysis for upstream regulators of the gene expression response to radiation in the two strains and found specific regulators of radiation response and immune function showing similar activity trends in the two genotypes. Significant activation of TP53, LPS, STAT1, IRF3 and IKBKB was indicated in the gene expression response of both mouse types. However, BNIP3L, IL27 and BCL2L1 showed opposite trends of activity in the two genotypes (Fig. 5b). We also identified a set of pathways and regulators that appeared significant in one genotype but not the other. These may suggest specific differences in the radiation response of WT and *Il10*^*−/−*^ mice.

### Impact of inflammatory response on prediction of radiation exposure

Since one of our broad objectives is the development of gene expression signatures predictive of radiation exposure, we used microarray data to build and test classifiers using a greedy pairs method with 7 different algorithms. To test the ability of a gene set selected from WT data to identify radiation exposure in the presence of chronic inflammation, a set of 24 genes was identified using a greedy pairs algorithm and the WT samples as a training data set (Table 2). The data from the *Il10*^*−/−*^ mice were then used as a test set, and all 7 algorithms predicted the exposure status correctly with 100% accuracy (*P*-value 0.05). Many of the genes selected in the classifier were well-established radiation responsive genes with roles in the Trp53 pathway or in inflammation and immune response*.* In fact, “Immunity” was the only GO category significantly overrepresented (Benjamini corrected *p*-value = 0.025 by DAVID analysis) among the selected signature genes. As genes with immune functions formed such a significant part of the signature, we also used the *Il10*^*−/−*^ data to train a classifier (Table 3) and tested it on the WT data to see what impact this would have. The new classifier included a higher proportion of immunity related genes (“Immunity” overrepresented with a Benjamini corrected p-value = 0.0019 by DAVID analysis), and performed with 100% accuracy on the *Il10*^*−/−*^ samples during cross validation. Despite the increased contribution from immunity related genes, all 7 algorithms also predicted the exposure status of the WT test samples correctly with 100% accuracy.

**Table 2:** Composition of 24-gene classifiers distinguishing radiation exposure. Features were selected using the greedy pairs method using WT mouse data as the training set

	UniqueID	Symbol	Name	Parametric *p*-value	Geom mean of intensities in Irradiated	Geom mean of intensities in Controls	Fold-change (Irradiated/controls)
1	A_51_P391955	Dapl1	death associated protein-like 1	< 1e-07	5.74	354.08	0.016
2	A_55_P2136906	Vpreb3	pre-B lymphocyte gene 3	< 1e-07	23.78	952.67	0.025
3	A_55_P2082929	H2-Ob	histocompatibility 2, O region beta locus	< 1e-07	25.11	544.45	0.046
4	A_51_P378298	Fcmr	Fc fragment of IgM receptor	< 1e-07	93.84	2574.17	0.036
5	A_55_P2108943	Ccr6	chemokine (C-C motif) receptor 6	< 1e-07	9.01	390.03	0.023
6	A_55_P2117614	Tnfrsf13c	tumor necrosis factor receptor superfamily, member 13c	< 1e-07	18.07	470.05	0.038
7	A_55_P1958275	Bcl11a	B cell CLL/lymphoma 11A (zinc finger protein)	< 1e-07	14.18	304.79	0.047
8	A_55_P2332731	D130062J21Rik	RIKEN cDNA D130062J21 gene	< 1e-07	23.24	302.25	0.077
9	A_55_P1964648	Btla	B and T lymphocyte associated	< 1e-07	40.07	445.85	0.09
10	A_66_P130916	H2-Ob	histocompatibility 2, O region beta locus	< 1e-07	54.33	623.04	0.087
11	A_52_P417654	Tcea1	transcription elongation factor A (SII) 1	1.6e-06	54.29	229.5	0.24
12	A_66_P103490	Srsf1	serine/arginine-rich splicing factor 1	5.8e-06	34	88.08	0.39
13	A_51_P292030	Nus1	NUS1 dehydrodolichyl diphosphate synthase subunit	0.0009584	197.92	116.11	1.7
14	A_51_P223443	Cd81	CD81 antigen	8.2e-06	7009.38	2854.95	2.46
15	A_55_P2145804	Aen	apoptosis enhancing nuclease	4.6e-06	909.81	172.36	5.28
16	A_55_P1952379	Fkbp5	FK506 binding protein 5	3.3e-06	678.47	229.64	2.95
17	A_55_P1998943	Oas1a	2′-5′ oligoadenylate synthetase 1A	5e-07	5226.79	408.46	12.8
18	A_52_P612803	Ccng1	cyclin G1	3e-07	423.04	79.12	5.35
19	A_52_P90363	Ifi27l2a	interferon, alpha-inducible protein 27 like 2A	1e-07	30,011.04	2971.34	10.1
20	A_51_P154842	Oas1f	2′-5′ oligoadenylate synthetase 1F	< 1e-07	2053.56	233.96	8.78
21	A_51_P363947	Cdkn1a	cyclin-dependent kinase inhibitor 1A (P21)	< 1e-07	897.24	24.09	37.24
22	A_55_P1972872	I830012O16Rik	RIKEN cDNA I830012O16 gene	< 1e-07	462.01	18.35	25.17
23	A_51_P259975	Aspa	aspartoacylase	< 1e-07	159.62	10.1	15.8
24	A_51_P329928	Phlda3	pleckstrin homology like domain, family A, member 3	< 1e-07	2163.11	137.61	15.72

**Table 3:** Composition of 24-gene classifiers distinguishing radiation exposure. Features were selected using the greedy pairs method using *Il10*^−/−^ mouse data as the training set

	UniqueID	Symbol	Name	Parametric p-value	Geom mean of intensities in Irradiated	Geom mean of intensities in Controls	Fold-change (Irradiated/Controls)
1	A_55_P2108943	Ccr6	chemokine (C-C motif) receptor 6	< 1e-07	11.77	554.18	0.021
2	A_51_P248122	Bbc3	BCL2 binding component 3	< 1e-07	1019.01	263.9	3.86
3	A_51_P378298	Fcmr	Fc fragment of IgM receptor	< 1e-07	41.34	1536.63	0.027
4	A_51_P304859	Zfand6	zinc finger, AN1-type domain 6	4e-07	115.94	422.84	0.27
5	A_51_P371750	Marco	macrophage receptor with collagenous structure	< 1e-07	5630.68	341.13	16.51
6	A_52_P612803	Ccng1	cyclin G1	< 1e-07	483.89	90.38	5.35
7	A_51_P363947	Cdkn1a	cyclin-dependent kinase inhibitor 1A (P21)	< 1e-07	1154.64	47.87	24.12
8	A_55_P2121856	Ier5l	immediate early response 5-like	< 1e-07	172.96	53.17	3.25
9	A_55_P2137496	Cd79a	CD79A antigen (immunoglobulin-associated alpha)	< 1e-07	138.15	1777	0.078
10	A_51_P205779	Cd5l	CD5 antigen-like	6e-07	8412.39	1257.39	6.69
11	A_51_P329928	Phlda3	pleckstrin homology like domain, family A, member 3	< 1e-07	2635.66	186.27	14.15
12	A_52_P77080	Gypa	glycophorin A	< 1e-07	93.67	1314.36	0.071
13	A_55_P2007964	Cx3cr1	chemokine (C-X3-C motif) receptor 1	< 1e-07	46.72	374.07	0.12
14	A_55_P2051254	Pvt1	plasmacytoma variant translocation 1	< 1e-07	118.94	27.32	4.35
15	A_51_P327751	Ifit1	interferon-induced protein with tetratricopeptide repeats 1	< 1e-07	545.07	82.18	6.63
16	A_51_P226429	Rhobtb2	Rho-related BTB domain containing 2	1.2e-06	51.68	128.33	0.4
17	A_51_P359570	Ifit3	interferon-induced protein with tetratricopeptide repeats 3	< 1e-07	2195.72	75.33	29.15
18	A_52_P497625	A630001G21Rik	RIKEN cDNA A630001G21 gene	1.4e-06	40.17	113.97	0.35
19	A_66_P120987	Cd79a	CD79A antigen (immunoglobulin-associated alpha)	< 1e-07	21.65	180.7	0.12
20	A_55_P2105256	Trim47	tripartite motif-containing 47	< 1e-07	373.14	76.03	4.91
21	A_55_P1958275	Bcl11a	B cell CLL/lymphoma 11A (zinc finger protein)	< 1e-07	9.03	228.33	0.04
22	A_52_P180565	2310061I04Rik	RIKEN cDNA 2310061I04 gene	3.3e-06	158.13	373.06	0.42
23	A_52_P390944	Chst3	carbohydrate (chondroitin 6/keratan) sulfotransferase 3	< 1e-07	8.68	274.93	0.032
24	A_55_P2097474	Creb5	cAMP responsive element binding protein 5	6.6e-06	1535.01	3450.09	0.44

**Fig. 6:**
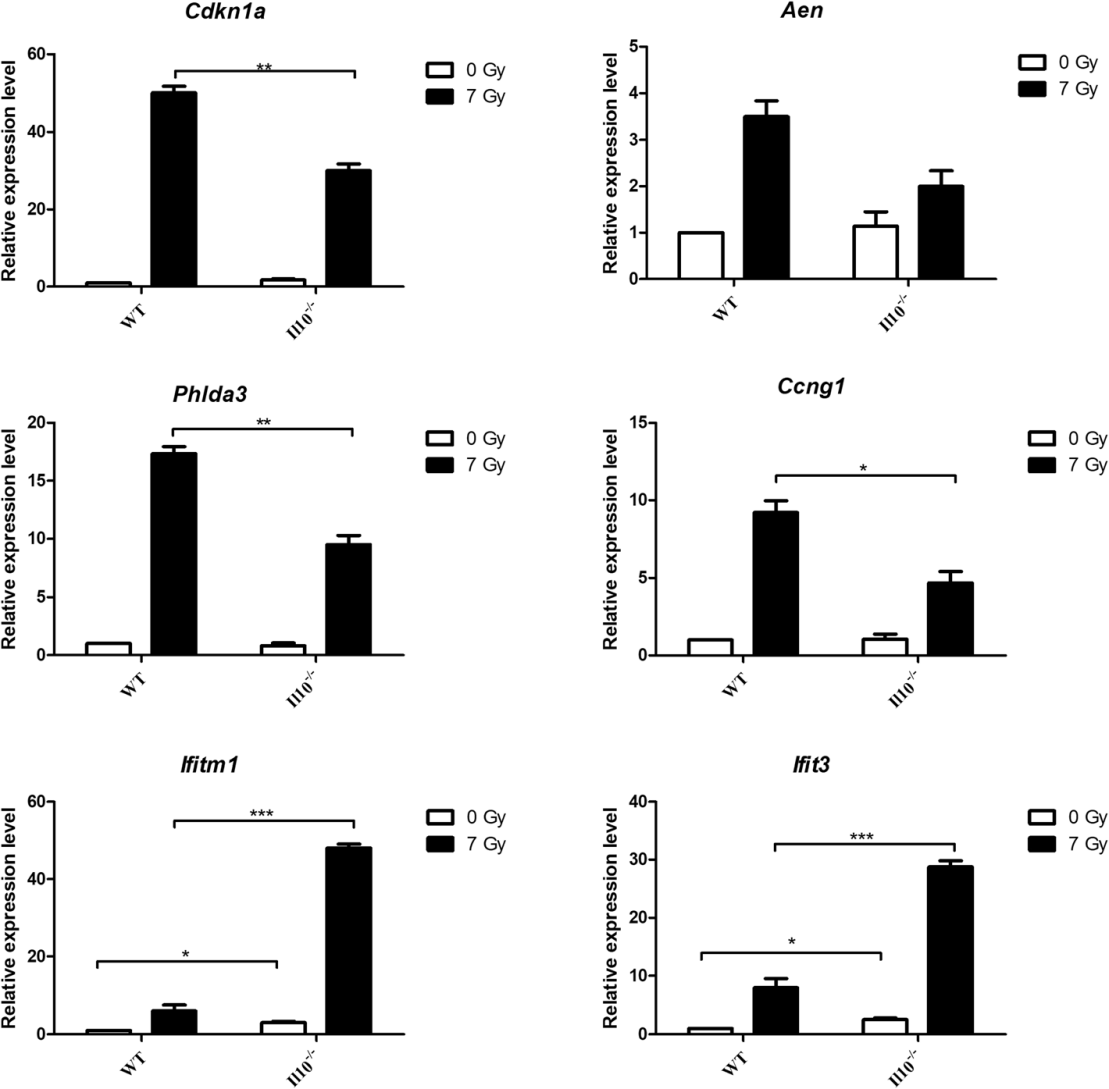
Gene expression measured by qRT-PCR. Expression levels of 6 genes (*Cdkn1a, Aen, Phlda3, Ccng1, Ifitm1, Ifit3)* showing significant difference in expression in *Il10*^−/−^ mice compared to WT after radiation exposure are presented here. The expression level for each gene was calculated relative to the expression levels in unexposed WT animals considered as 1. The expression was normalized against Beta-Actin gene expression. The data is represented as mean +/− SEM (*n* = 5). * Significant at *P* < 0.05, ** Significant at *P* < 0.01, *** Significant at *P* < 0.001, using unpaired t-test

Finally, we validated expression levels of six of the classifier genes using quantitative real-time PCR. We chose four Trp53-responsive genes, *Cdkn1a, Aen, Ccng1* and *Phlda3 and* two inflammatory genes, *Ifit1* and *Ifit3,* for this analysis. As observed in the microarray results, we found a greater relative fold change of Tp53-responsive genes in WT compared to *Il10*^*−/−*^ mice after radiation exposure, and a greater relative fold change of inflammatory cytokines in *Il10*^*−/−*^ mice (Fig. 6). The inclusion of a 2 Gy dose measurement of *Cdkn1a, Phlda3, Ifitm1* and *Ifit3* (Additional file 6) suggests that the relative responses of the Trp53 regulated genes in the two strains are similar at low and high doses. In contrast, the heightened response of inflammatory genes in the *Il10*^*−/−*^ mice, although still significant for *Ifitm1* at 2 Gy, was much greater at the higher dose. A more detailed delineation of the interaction between radiation dose and inflammatory gene expression in these two genotypes would be of interest.

## Discussion

Exposure to ionizing radiation has long been known to cause production of various immune molecules like cytokines, DAMPs (Damage-associated molecular patterns) and cellular factors in the irradiated tissue, and to result in inflammation [5, 6]. The degree of radiation mediated inflammation varies in individuals and depends on various factors including genetic, life-style and pre-existing disease conditions [14]. Inflammation has been indicated as a potential confounding factor in the development of IR biomarkers in epidemiological studies [31]. Previous transcriptomic studies have shown that immune and inflammation-related genes dominate the gene expression responses seen after in vivo radiation exposure [17, 31] and therefore, inflammation related genes have been proposed as biomarkers of radiation exposure or to be used in biodosimetric panels [32]. This raises the concern that inflammation may affect radiation responses, and may impact the use of some genes in biodosimetric panels for use in triage after an accidental or deliberate radiological event. Several investigators have used treatment with LPS, a general component of the bacterial cell wall that is a potent inducer of inflammatory response [30, 31], as a model for acute inflammation or infection. LPS has been shown to induce expression of some well-established radiation responsive genes and pathways [33–35], furthering concern about the impact of inflammatory states. As an initial approach to examining the potential impact of a pre-existing chronic inflammatory condition on radiation biodosimetry, we have used a well-established mouse model (*Il10*^−/−^) of human chronic inflammatory bowel disease (IBD) and compared its gene expression with and without radiation exposure with that of wild type (WT) littermates.

First, we compared blood counts in the two genotypes before and after irradiation. Of note was the significant difference in the baseline level of neutrophils in *Il10*^−/−^ compared to WT mice. Neutrophils are generally associated with inflammatory response. In addition, a higher level of neutrophils has been reported in IBD patients, which correlates with the severity of their clinical manifestations [36, 37]. The higher level of neutrophils in *Il10*^−/−^ mice is similar to that in individuals suffering from IBD. The increased proportions of neutrophils contribute to a pro-inflammatory blood immune environment, which creates a concern of possible false positives or interference with radiation response for dosimetric biomarkers.

We next compared the baseline gene expression levels in un-irradiated *Il10*^−/−^ mice with those in WT mice (Fig. 2). The genes that showed significant differences in expression were mainly involved in inflammatory immune response, as might be expected, as well as glutathione metabolism and collagen deposition. In a recent publication, Mak et al. [38] showed that glutathione metabolism plays an important role in the activation process of inflammatory T-cells in response to immune stimuli. Excessive deposition of collagen, a major constituent of the extracellular matrix, has also been associated with inflammation and the development of fibrosis [39], functions also associated with radiation exposure. We found LPS / IL-1 signaling, a major regulator of gene expression responses in IBD patients [40], to be a significant pathway connecting the processes of inflammation, glutathione metabolism and fibrosis (Fig. 2c). These results suggest that chronic inflammatory conditions can indeed trigger some of the same gene expression patterns indicative of radiation response in the absence of exposure.

Transcriptomic analysis of radiation responses in the two genotypes revealed broad similarity among the gene ontology terms most significantly affected by radiation exposure. Unsurprisingly, these included p53 signaling and some immune related functions (Fig. 4). Although significantly enriched among radiation responsive genes in both genotypes, the canonical radiation response of p53 signaling did appear to be slightly muted in the *Il10*^−/−^ mice, as implied by a slightly higher *P* value compared to WT-mice. This suggests that fewer p53-regulated genes responded significantly to radiation in the mutant mice. In confirming the radiation induction of individual genes, we found that both *Cdkn1a* and *Aen* were up regulated by radiation exposure to a lesser degree in the *Il10*^−/−^ mice, compared to WT mice (Fig. 6). There is some evidence in the literature of a regulatory link between IL10 and p53 in inflammatory processes. IL10-induced apoptosis in mouse bone marrow-derived mast cells has been shown to depend on activation of p53 [41]. Levels of IL10 and p53 were also found to be correlated in patients with autoimmune disease, with high levels of IL10 apparently leading to overexpression of p53 [42]. Taken together, these observations suggest that IL10 may contribute to the regulation of p53, leading to a blunted radiation response of genes downstream of p53 in the absence of IL10.

The observed enrichment of genes with functions related to B-cell development among the down-regulated genes of both genotypes could be explained by the observed overall decrease in lymphocyte levels after radiation exposure (Fig. 1), particularly as B cells are among the most radiosensitive hematopoietic cells [43].

We observed that specific immune functions like MHC based antigen presentation and interferon signaling were particularly associated with radiation responsive genes in *Il10*^*−/−*^ mice but not WT mice. Antigen presentation mainly occurs through major histocompatibility complex (MHC) receptors on the cell surface, and is a very important process in activation of T-cells, which mount an immune response by releasing cytokines or protein mediators of apoptosis [44]. The MHC-I is present on the cell surface of all the nucleated cells while MHC-II is present only in specific antigen presenting cells (APC), like macrophages and dendritic cells. Antigen presentation through MHC-II receptors results in activation of T-helper (Th) cells, which modulate the immune environment by releasing specific cytokines [44, 45]. We found genes involved in MHC-II based antigen presentation to be specifically downregulated by radiation exposure in WT mice but upregulated in *Il10*^*−/−*^ mice. This indicates a differential modulation of the antigen presentation mechanism and its downstream immune response in the two genotypes after radiation exposure.

It has been reported that similar to IBD patients, *Il10*^*−/−*^ mice have higher levels IL23 producing macrophages, which through their interaction with T-cells via MHC-II receptors cause production of various inflammatory cytokines [25]. Intriguingly, IPA predicted a significant response to radiation of T-cell signaling in both genotypes, but in opposite directions. In *Il10*^−/−^ mice the gene expression pattern suggested a significant radiation activation of T-cell response, while in WT mice this pathway was predicted to be inhibited. Similarly, the T-helper type-1 (Th-1) immune response pathway was predicted to follow the same response pattern, with the pathway appearing to be activated by radiation exposure in *Il10*^−/−^ mice and inhibited in WT mice. Th1 mediated signaling has been reported to be the major causes of inflammation in people suffering from IBD as well as in animal models of IBD [24, 40, 45]. Production of interferons like IFN-gamma is a hallmark of Th1 immune response, and is also required for driving T-helper cell populations towards the Th1 type of immune response [46]. We found significant upregulation of interferon and its associated genes in *Il10*^*−/−*^ mice but not in WT after radiation exposure. This type of immune response also plays an important role against viral and bacterial infection. We found a significant association of these specific gene ontology terms specifically with the radiation response in *Il10*^*−/−*^ mice (Fig. 4; Additional file 5). Overall, we found that radiation exposure results in higher inflammatory responses in *Il10*^*−/−*^ mice compared to WT, and this appears to be mediated by the T-cell population.

### Impact on biodosimetry markers

Since our laboratory is interested in the development of biodosimetry markers for radiation exposure, we also used our data to select and test panels of genes that could distinguish radiation exposure in individual samples regardless of pre-existing inflammatory disease. The panels included p53 responsive genes like *Cdkn1a* and *Aen* as well as immune responsive genes like *Ifit1* and *Ifit3*. *Cdkn1a* expression is known to respond widely to a number of stresses, and has been previously shown to respond to the inflammation inducing agent LPS [32]. In our current study, we found a significant difference between WT and *Il10*^−/−^ expression levels of *Cdkn1a* and other p53 regulated genes after radiation exposure, with the *Cdkn1a* radiation response being significantly muted in *Il10*^−/−^ mice compared to WT. The reverse was true of several immune related genes, which showed a larger magnitude of response to radiation exposure in the *Il10*^−/−^ mice compared to WT mice. Although in our current study these differences in response did not limit our ability to detect a relatively damaging dose of radiation, these results do suggest that excessive reliance on genes in these pathways may have an impact on the ultimate goal of dose discrimination. Care must also be taken to ensure that the elevated expression of inflammatory genes noted in the unirradiated *Il10*^−/−^ mice does not lead to misidentification of such inflammation-prone individuals as having received a small dose of radiation. Future studies across a range of doses will be needed to fully understand the impact of preexisting inflammation states on radiation biodosimetry using gene expression.

In a previous study, we had shown that genes selected from WT mice performed poorly in detecting exposure to an LD_50/30_ radiation dose in two mouse models of DNA repair deficiency (*Atm*^*−/−*^ and *Prkdc*^*scid*^), but that inclusion of both WT and DNA repair deficient mice in the training set improved performance on both mutant and WT test sets to 100% [47]. We tested the ability of this earlier signature to predict radiation exposure in *Il10*^−/−^ mice. Although only one gene, *Cdkn1a*, was common between the earlier signature and either of the signatures reported in this study, we tested the WT plus repair mutant gene set on the dataset from this study and found that it was again able to predict the radiation exposure in *Il10*^−/−^ mice, and the new set of WT mice, with 100% efficiency (*p* < 0.005). This supports inclusion of individuals with a range of radiation sensitivities and underlying disease states in gene selection to produce more robust signatures for radiation biodosimetry.

## Conclusions

Using a mouse model of inflammatory bowel disease, we have found that a chronic inflammatory state can impact both the basal expression levels and the relative radiation response of genes involved in immune and inflammatory pathways, as well as other key radiation response pathways, such as p53. While this did not impact the ability of gene expression signatures derived from either WT or pro-inflammatory *Il10*^−/−^ mice to correctly distinguish between unexposed and irradiated mice, it does raise concerns for the accurate determination of doses across a broad dose range. Future experiments employing multiple radiation doses will enable selection of genes with expression levels unaffected by underlying inflammation, and will help to avoid confounding of radiation biodosimetric gene expression signatures by underlying chronic inflammation.

## Additional files

Additional file 1: Total white blood cell counts in WT and *Il10*^−/−^ mice after 2 Gy of radiation exposure A) Counts at Day1 post exposure B) Counts at Day 30 post exposure. Green circles (control) and red squares (irradiated) indicate values for individual animals of the respective genotype. Significant differences *P < 0.05 (*n* = 8). (JPG 360 kb)

Additional file 2: A. Genes showing significant differences in expression levels between WT and *Il10*^−/−^ mice prior to radiation exposure**.** B. Gene ontology (GO) analysis genes using DAVID [30]. Top 5 significant (Benjamini corrected *P*-value< 0.05) GO terms are shown in the bar diagram. C. IPA based network analysis. Top 10 significant (*P* < 0.05) Canonical pathways are shown in bar diagrams. (XLSX 323 kb)

Additional file 3: Real-time qRT-PCR validation of 5 genes (*Gstt1, Il18r1, Alas1, Acsbg1, Cyp2b6*) relevant to the current study, showing significant difference in expression at baseline level between WT and *Il10*^−/−^ mice prior to radiation exposure. The data is represented as mean +/− SEM (*n* = 5) for each genotype. * Significant at *P* < 0.05 using unpaired t-test. (JPG 398 kb)

Additional file 4: BRB-array tools output of differentially expressed genes after radiation exposure in the two genotypes. Results of each genotype are in a separate tab. (XLSX 708 kb)

Additional file 5: IPA network analysis of genes involved in T-cell signaling in A. WT and B. *Il10*^−/−^ mice in response to radiation. Statistical z-score (> 2 or < − 2) was used for prediction of the activation status of the signaling pathway. Orange lines indicate activation (z ≥ 2) and blue indicates inhibition (z ≤ − 2), Grey lines indicate no significant contribution to activity. Up-regulated genes are shown in red and down-regulated genes in green. (JPG 165 kb)

Additional file 6: Dose response curves of expression of 4 genes (*Cdkn1a, Phlda3, Ifitm1, Ifit3*) based on qRT-PCR analysis. Relative gene expression at 0, 2 and 7 Gy of radiation exposure is shown for WT (dotted line) and *Il10*^−/−^ mice (Solid line). Relative expression was calculated compared to the expression level at 0 Gy (unexposed-sham controls) of WT considered as 1. The expression was normalized against Actin-Beta gene expression. The data is represented as mean +/− SD (*n* = 5) for each genotype. * Significant at *P* < 0.05, **Significant at *P* < 0.01, *** Significant at *P* < 0.001, using unpaired t-test. (JPG 598 kb)

## Abbreviations

DAVID: Database for annotation, visualization and integrated discovery
FDR: False discovery rate
GO: Gene ontology
IBD: Inflammatory bowel disease
IPA: Ingenuity pathway analysis
qRT-PCR: quantitative Real-Time Polymerase Chain Reaction
