# Supplementary figures and images for: Impact of inflammatory signaling on radiation biodosimetry: mouse model of inflammatory bowel disease
Source: BMC Genomics. 2019 May 2;20:329. doi: 10.1186/s12864-019-5689-y (PMC6498469; doi:10.1186/s12864-019-5689-y)

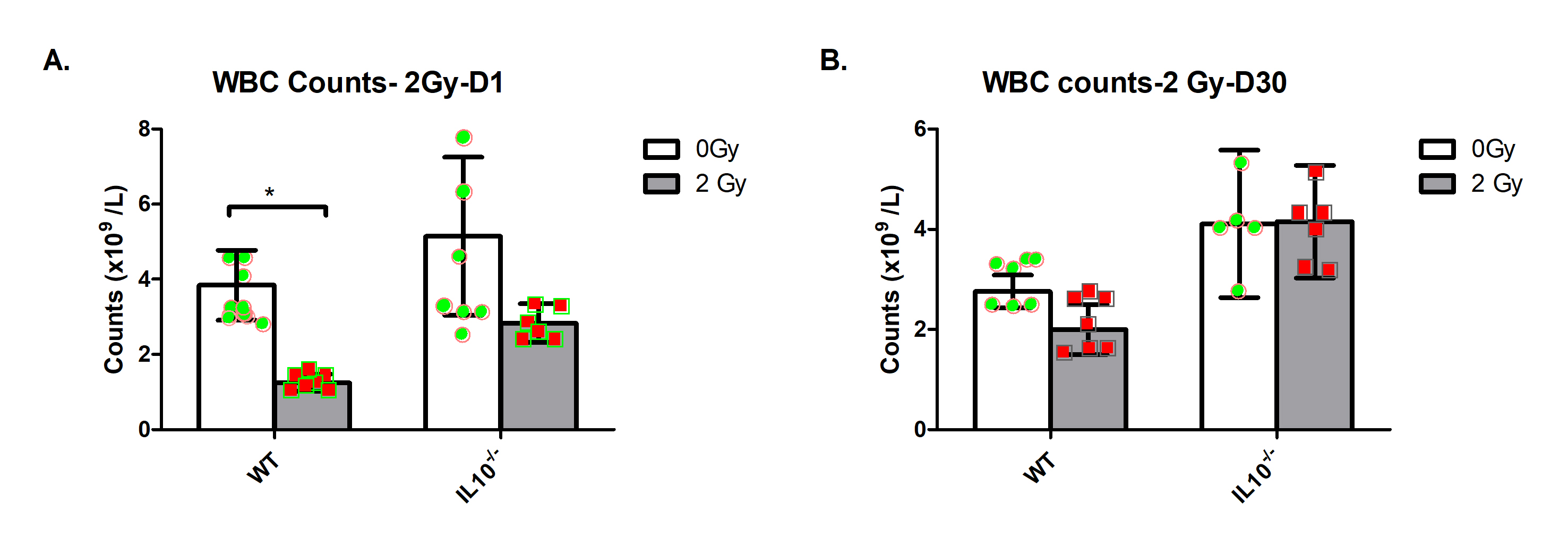

Supplement: Supplementary file 1 — Total white blood cell counts in WT and Il10−/− mice after 2 Gy of radiation exposure A) Counts at Day1 post exposure B) Counts at Day 30 post exposure. Green circles (control) and red squares (irradiated) indicate values for individual animals of the respective genotype. Significant differences *P < 0.05 (n = 8). (JPG 360 kb) [file 12864_2019_5689_MOESM1_ESM.jpg]

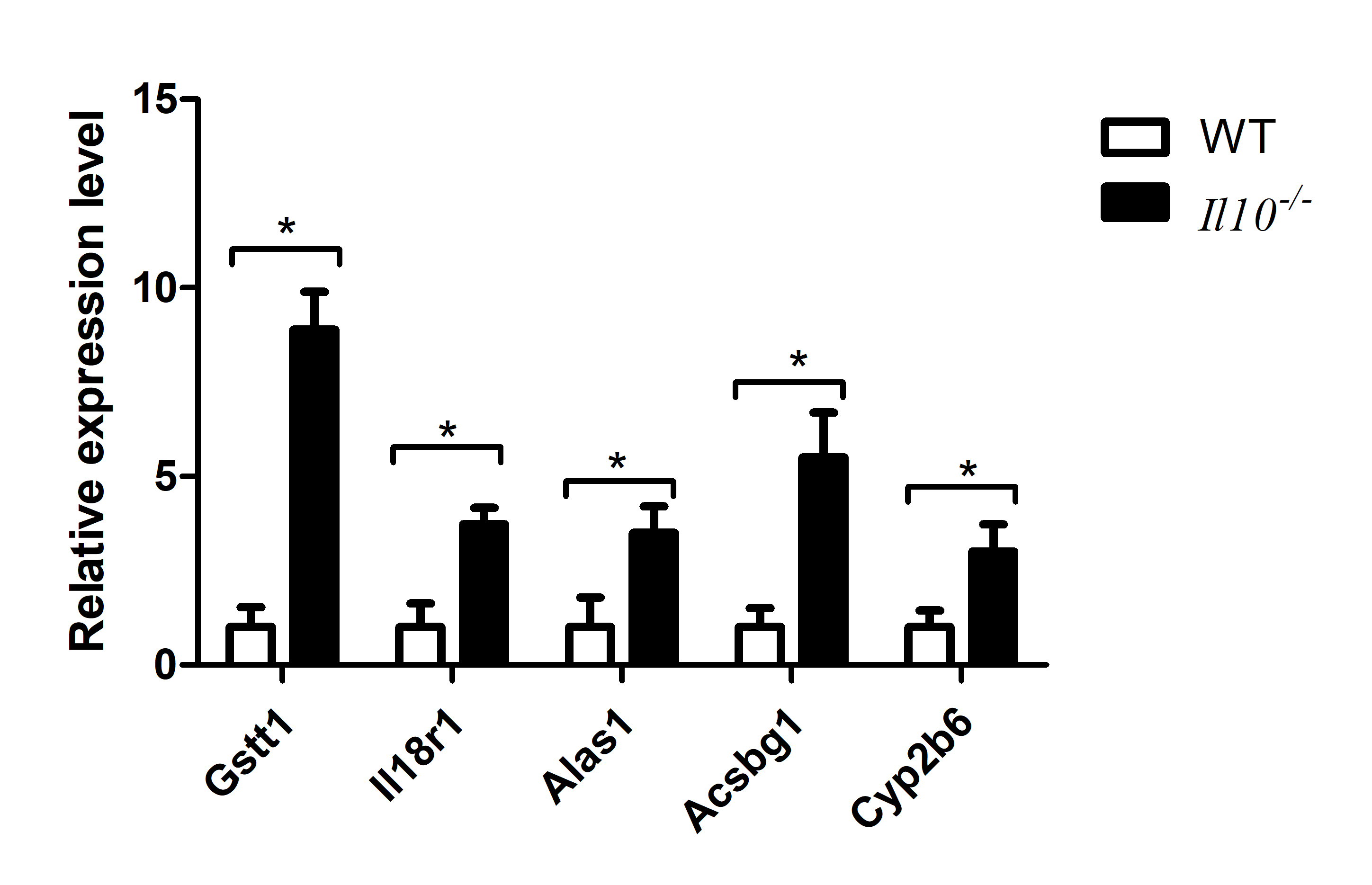

Supplement: Supplementary file 3 — Real-time qRT-PCR validation of 5 genes (Gstt1, Il18r1, Alas1, Acsbg1, Cyp2b6) relevant to the current study, showing significant difference in expression at baseline level between WT and Il10−/− mice prior to radiation exposure. The data is represented as mean +/− SEM (n = 5) for each genotype. * Significant at P < 0.05 using unpaired t-test. (JPG 398 kb) [file 12864_2019_5689_MOESM3_ESM.jpg]

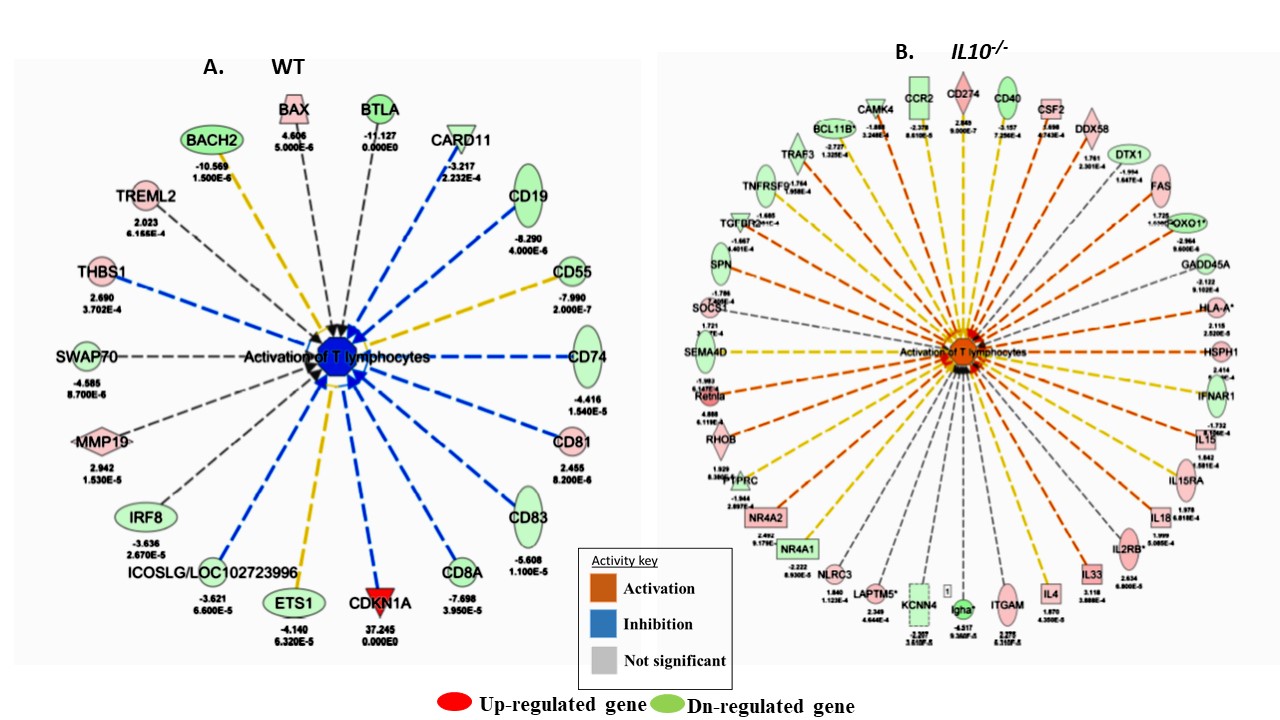

Supplement: Supplementary file 5 — IPA network analysis of genes involved in T-cell signaling in A. WT and B. Il10−/− mice in response to radiation. Statistical z-score (> 2 or < − 2) was used for prediction of the activation status of the signaling pathway. Orange lines indicate activation (z ≥ 2) and blue indicates inhibition (z ≤ − 2), Grey lines indicate no significant contribution to activity. Up-regulated genes are shown in red and down-regulated genes in green. (JPG 165 kb) [file 12864_2019_5689_MOESM5_ESM.jpg]

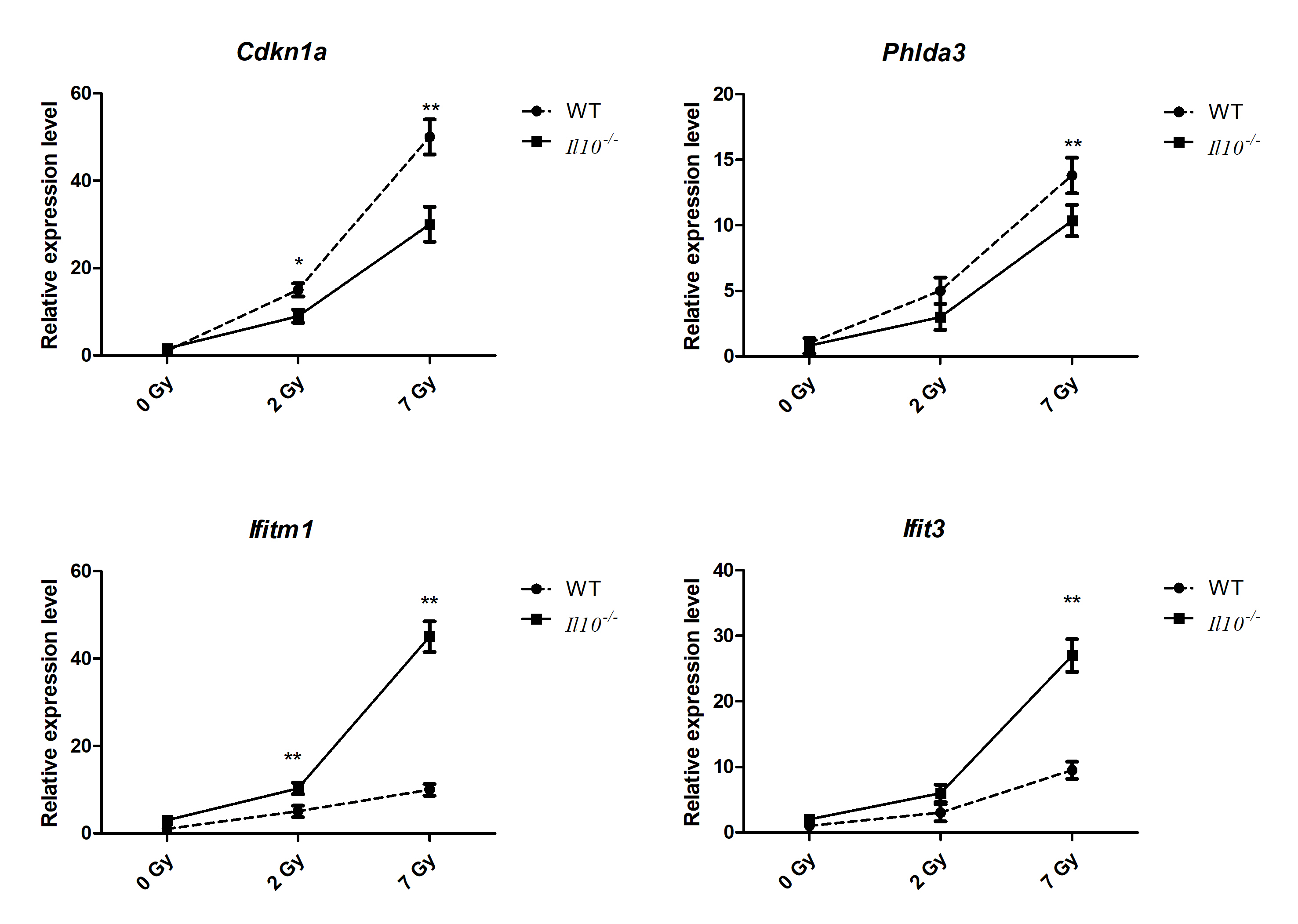

Supplement: Supplementary file 6 — Dose response curves of expression of 4 genes (Cdkn1a, Phlda3, Ifitm1, Ifit3) based on qRT-PCR analysis. Relative gene expression at 0, 2 and 7 Gy of radiation exposure is shown for WT (dotted line) and Il10−/− mice (Solid line). Relative expression was calculated compared to the expression level at 0 Gy (unexposed-sham controls) of WT considered as 1. The expression was normalized against Actin-Beta gene expression. The data is represented as mean +/− SD (n = 5) for each genotype. * Significant at P < 0.05, **Significant at P < 0.01, *** Significant at P < 0.001, using unpaired t-test. (JPG 598 kb) [file 12864_2019_5689_MOESM6_ESM.jpg]
